# Dr. Pearson, on the Inoculated Vaccina

**Published:** 1801-01

**Authors:** George Pearson

**Affiliations:** Leicester Square


### *To the Editors of the Medical and Phyſical Journal.*

Miſtaque cum veris paſſim commenta vagantur

Millia rumorum: confuſaque verba volutant.

GENTLEMEN,

THE rumours concerning the diſcreditable caſes of the inoculated Cow-pock lately at Clapham, were ſuch as to excite the attention, and provoke the inquiry, of perſons even little intereſted in the fortunes of the new practice: But from the part I have taken in attempting to introduce the Vaccine Inoculation, I found myſelf particularly called upon to relate ſome

---

ſome particulars, and to aſſign reaſons for thoſe unfavorable occurrences. I confeſs I was much diſappointed to find that the Report publiſhed in your laſt Journal, not only did not ſatisfy my mind with reſpect to the aſſigned cauſe of theſe unfavorable inſtances, but contained ſeveral aſſertions of great practical importance, which are at variance with the evidence of my own experience. Hence I thought it would be culpable conduct on my part, to withhold from the Public the adverſe evidence which I poſſeſs, ſincerely hoping that the communication of it will be beneficial, whether the concluſions in the Report be invalidated or not.

The following paragraph contains the moſt important aſſertions, which principally induced me to come forward on the preſent occaſion. "The gentleman," I am authorized to ſay, Mr. Buckland, "who performed this inoculation, candidly acknowledges, that the matter with which he had charged the lancet for this purpoſe, had been taken at a very late period of the diſeaſe, after a brown ſcab had formed, and had a purulent appearance at the time it was taken from the arm, conſequently had, by degenerating, loſt the power of giving the Cow-pox infection, and appears to have been the cauſe of all the miſchief that enſued."

Now, as the concluſions in the Report are principally built upon the kind of *Matter* employed, it is to my apprehenſion needful to aſcertain with preciſion, the origin, and many of the properties and circumſtances of this inoculated *Matter*, to which ſo much miſchief is imputed. Accordingly I find, on the teſtimony of the Inoculator,

1. That it was taken on the lancet, uſed in the firſt inoculation mentioned in the report, from two of the patients of that inoculation on *the tenth day*, and who underwent the diſeaſe favorably. This lancet had not been cleaned ſubſequently to the firſt inoculation, but it was not ruſty, and the matter on it was inſerted while fluid, within an hour after it was taken; and no other matter was taken on this lancet employed in the inoculation, but that from the two patients, as aſſerted moſt diſtinctly and uniformly to me by Mr. Buckland in three different converſations.

2. The matter uſed in the firſt inoculation, may be ſafely aſſumed to have been that of the genuine Vaccine pock, from the words of the Report, namely, "that there were no appearances in any degree deviating from the progreſs of the Cow-pox." It is however, I think, material to remark, that Mr. B's ſtatement informs me that one of the patients of the firſt inoculation had unfavorable ſymptoms, like thoſe of the ſecond inoculation, and that in ſeveral of the patients unfavorable ſymptoms toms fupervened foon after taking matter with the lancet on the tenth day, for the fecond Inoculation.

3. It is next in order in this inveftigation, to inquire into the fource of the matter inferted in the firft inoculation, which was performed on Wednefday, October 22. Here we find that Mr. B. collected matter on a *lancet*, from a patient at Clapham, under the care of Mr. L.* an eminent Practitioner in the city, on *the twelfth day* after inoculation; the patient having been inoculated on Saturday, October 11, and it was employed while fluid, within an hour after it had been obtained, and the Report ftates, on "fifteen perfons, but only fix took the infection."

4. It is of importance to be known, that Mr. L. above alluded to, on Thurfday, October 23, *the thirteenth day* after inoculation, a day later than Mr. B. "finding," fays he, "the inflammation round the puftule ftill continuing bright, and *the fluid very thin*, I took a little on the point of a lancet for Mr. D. which not being fcarcely vifible, he doubted of fuccefs; with this he inoculated a child on Saturday morning, (November 1): The inoculation fucceeded, and numbers have been fince inoculated from this fource, and one is under my care at this time, the eighth day." This laft named patient was obligingly fhown to me by Mr. L. on Saturday, December 13, then the thirteenth day after inoculation, having the ufual appearances of the vefìcle in the incipient ftate of deficcation.

I am under the neceffity of delivering evidence from my own obfervation, which contravenes the conclufion drawn in the Report; that as matter was taken late in the difeafe, and after a brown fcab had formed, which had a purulent appearance, it "*consequently had, by degenerating, lost the power of giving the Cow-pock infection.*" Now my own practice, as well as that of fome of my friends, inftructs me, that purulent matter formed in a genuine Vaccine pock, not later than the 12th day after inoculation, can produce the genuine mild *Vaccina.* If analogy were needful to eftablifh this truth, I would appeal to the practice of the variolous inoculation; in whitch, I believe, no Inoculator hefitates to ufe purulent, as well as limpid, matter. However I do not mean to fay, that it is proved that purulent vaccine fluid is equally efficacious as the limpid fluid; for fuppuration fo feldom takes place, that I have not facts to prove more than the harmleffnefs and efficacy of fuch matter where I have ufed it; but confequently, that purulency does not neceffarily imply, as afferted, *degeneration.*

To

* This patient of Mr. L. was inoculated with matter of the tenth day, from Mr. L———s', the furgeon, child.

To prove that the *Matter* ufed by Mr. B. was the occafion of the anomalous vaccine cafes, it is ftated in the Report, "that in two of the patients who are believed to have had the Cow-pock diftinctly by the former inoculation, the fame fymptoms were unfortunately induced in attempting to procure Cow-pox fluid from them." If the lancet was in the condition I have above ftated, I prefume to think that the enfuing inflammation, at leaft partly, arofe from the incifions, or would have taken place, as is frequently the cafe, when the veficle begins to dry, independent of any external irritation. Nor is this opinion difcountenanced, but rather confirmed, by the *great proportion* of fimilar anomalous cafes in the late inoculation; for examples are wanting of a particular morbific matter, which will produce on healthy parts the effects on the places inoculated fo generally as related in the Report.*

Another fact, afferted on good authority, inclines me to the opinion, that if venomous matter, different from the vaccine, were propagated, the vaccine was alfo mixed with it, is, that in one or more of the cafes a genuine vaccine veficle exifted on one arm, while on the other arm of the fame patient a confiderable ulceration took place.

With refpect to the five injunctions, as collected from a refpectable authority, in the Poftfcript of the Report, I fhall only take notice of two of them:

1. "That the Cow-pox *fluid* fhould be taken not later than the *ninth* day of the difeafe." This injunction was delivered, I believe, when the evidence of cafes of inoculated Cow-pock was extremely fcanty, comparatively with the ftate of it at this time, certainly in the proportion of 1 to 100. Among the great number of cafes which have fallen under my own obfervation, and from the ftill greater number which have been communicated to me, not a fingle unfavorable cafe, on the account of the matter being taken at any period between the feventh and thirteenth day, or even, in fome inftances, a day or two later, has been obferved. And it cannot efcape notice, that the cafes referred to in this paper, are evidences againft the injunction. The Regifters at the Vaccine Inftitution, which feem adequate to the determination of this point, fhow no difference in the degree, or kind of fymptoms of the inoculated *Vaccina*,

* As to the inflammation of the punctured part and conftitutional diforder which occurs fometimes in diffection, fuch a cafe does not happen, I believe, in more than one out of fifty or perhaps an hundred inftances of puncture; and on the very firft authority I am affured, it feems that the morbid effects arife fometimes from the punctures even with a clean needle or knife, independent of animal matter.

whether the matter was of the eighth or of the eleventh and twelfth days; fo that the Inoculators, as far as I know, give no preference. However, to fatisfy my own mind, in the opportunities which occurred after reading the Report, I made fome trials with precifion, by inoculating one arm of each patient with *Matter* of the feventh or eighth day, and the other with *Matter* of the twelfth day; but I could obferve no difference in the appearances of the Vaccine pock on each arm of the fame perfon. Among thefe cafes were two young children, who were inoculated, under my direction, by Mr. Donelly; and to prove that the matter was equally efficacious, I fhall ftate, that with matter, on the eighth day, from thefe patients, taken on twenty-fix lancets, and in the glafs bottles* with fpoon ftoppers, invented by Mr. Lewis, the apothecary to the Vaccine Inftitution, Mr. Carpue fuperintended the inoculation of forty-two perfons in the army; of whom, twenty-five took the difeafe, which they underwent with the ufual mild fymptoms, and without any perceivable difference in the fymptoms depending on the age of the matter.

With refpect to the fecond injunction, I have already had occafion to deliver the teftimony of my experience.

Whether the preceding evidence be fufficient to make out a Cafe manifefting that the anomalous and fevere fymptoms *did not depend on the Matter ufed having been taken fo late as the tenth day, from under a fcab, and allowing that it was purulent*, I fubmit to the decifion of the medical public: Nor is it within my prefent defign to bring into view other agents, which might occafion the bad fuccefs. I fhall only juft obferve if that had been my defign, I fhould particularly have pointed out the probable confequences of inoculating fo many prfons with *Matter on one lancet.* At the fame time it would have been but juftice, to confirm Mr. Buckland's depofition, by the extreme improbability of his taking *Matter* from any other fource but his own patients, on the tenth day. As to the gentlemen who have written the Report, I think it would be injuftice to their refpectable characters, to fuppofe an apology neceffary, where *the manifeftation of truth is the object.*

For the fatisfaction of fome perfons, it may be needful to obferve, that the prefent queftions ought not to be allowed the weight of a feather in argument againft the fafety and efficacy of the Vaccine Inoculation, although they are extremely important for determining on what the fuccefs depends, for avoiding hurtful agents, and for advancing knowledge of the animal œconomy. Nor will I, on any decifion of the points at iffue,

* Thefe bottles may be had at Meffrs. Parker's.

iſſue, recall an aſſertion which I have already made, that "a perſon during the inoculated Vaccina has a greater chance of life than under the ordinary circumſtances of living." But this propoſition is ſuppoſed only to be true, provided: 1. The Inoculator poſſeſs the knowledge already attained. 2. That he apply that knowledge with ſkill and ſedulous attention. 3. That he have the command over, or can avoid, the agency of thoſe powers which experience has ſhown to be hurtful. 4. That the patient be obedient to the directions preſcribed. Hence, however, it is manifeſt, that a patient may have a dangerous diſorder, and even die under the inoculated Cow pock, and yet the Practitioner be blameleſs. Although I very much doubt whether any perſon has ever died of the inoculated Vaccina, the above requiſites being preſent, it would be unwarrantable to affirm, that death may not take place either from the Cow-pock or from intervening diſorders during the Cow pock; for Phyſic, even in its preſent improved ſtate, cannot always "hold Mortality's ſtrong hand;"—it does not yet "bear the ſheers of Deſtiny."

As to one particular objection to the new Inoculation, on the ſcore of the origin of the venom in the Cow, and the offenſive aſpect of the eruption; if *uſefulneſs* be here the object of contemplation, a gem will be ſeen under a deformed exterior,

> "Which, like the toad, ugly and venomous,
> Wears yet a precious jewel in his head."

I am, yours, &c.

GEORGE PEARSON.

*Leiceſter Square, Dec.* 24, 1800.

P. S. In a Note to the Report under examination, the change which *Matter* undergoes in the Vaccine and Variolous puſtules, when it becomes virulent, is termed *decompoſition.* If this term had not an allowed definite import, and would not be likely from the authority of the writer to be employed in an unmeaning, vague ſenſe; or, what is worſe, according to its import; it would not be worth while to notice the impropriety of its uſe in this place. This impropriety will be moſt ſpeedily perceived by the queſtion, *What is the kind of change which takes place?* Now, I anſwer, without fear of contradiction, in what the change conſiſts is utterly unknown; but that as it is quite as reaſonable to *imagine* that it conſiſts in a new *compoſition* as in *decompoſition,* I beg to uſe the term *change* or *alteration,* and diſcard the term *decompoſition,* leſt a thing unknown, by the imagination bodied forth, obtain "a local habitation and a *name.*"

